# Assessing Technology Implementation Success for Highway Construction and Asset Management

**DOI:** 10.3390/s23073671

**Published:** 2023-03-31

**Authors:** Amit Tripathi, Gabriel B. Dadi, Hala Nassereddine, Roy E. Sturgill, Alexa Mitchell

**Affiliations:** 1 Department of Civil, Construction and Environmental Engineering, Iowa State University, Ames, IA 50010, USA; 2 Department of Civil Engineering, University of Kentucky, Lexington, KY 40506, USA; 3 HDR, Inc., Phoenix, AZ 85012, USA

**Keywords:** wireless technologies, technology maturity, implementation, people, process, technology, highway construction, asset management

## Abstract

The increasing demand for safe, reliable, and higher-quality infrastructure systems has led to more complex transportation construction and maintenance projects. This, coupled with the declining staff levels at many transportation agencies, requires a more comprehensive evaluation of technology implementation to compensate for these challenges. With a focus on effective technology implementation, this research goes beyond simply evaluating technologies to investigate technology implementation with personnel and policies at departments of transportation (DOTs). The study methodology involved a comprehensive literature review, a survey of all 50 state DOTs, and an in-person workshop of 18 DOT experts to validate the survey results and preliminary research findings. The findings support the need for those implementing technologies to understand people, processes, and technology maturity for their improved chances of implementation success. Using the approach presented, the DOTs can assess themselves and identify pathways to higher maturity levels in the areas of their people, processes, and technologies. This study also highlighted six factors that are important considerations for technology implementation and thus determined the relative importance of people, processes, and technology for these factors. The objective of this study was to assess the importance of people, processes, and technology that DOTs should prioritize to enhance the likelihood of successfully implementing technologies. The framework presented herein can be extended to any new or existing technology implementation initiatives at a DOT, including automatic identification and data capture (AIDC), emerging sensing and wireless technologies, safety technologies, and others.

## 1. Introduction

Technology implementation in highway construction and asset management has been an area of interest for researchers in the past few decades. The complex nature of transportation construction and asset management systems necessitates timely, accurate, reliable, and robust information. Research has shown that various technologies, including RFID, ultra-wideband tracking systems, GPS, augmented reality, building information modeling, e-ticketing, e-construction, digital twins, and others, can improve the construction and management of assets that are critical to highway agencies. There is a growing interest among the state departments of transportation (DOTs) in implementing emerging technologies, as demonstrated and supported by various Federal Highway Administration (FHWA) Every Day Counts (EDC) initiatives.

Technology can be leveraged within many areas of the DOT due to fewer transportation agency staff managing more lane miles and more complex projects [1,2]. However, the success of technology implementation efforts is not guaranteed. The variation in success is due to many factors, including regulations, funding, employee objections, management support, and more. This study proposes that these factors can be classified into people, process, or technology domains. Focusing on these three areas for successful technology implementation provides an opportunity to guide and support state DOTs during technology implementation. The motivation for this research comes from the growing digital transformation in transportation construction and asset management, which needs to deploy technology to complement and supplement the workforce successfully. This research argues that successful technology implementation requires a holistic review of the implementation efforts and dynamics collectively focused on people, processes, and technology.

Through a literature review, survey, interviews, and validation of metrics through an in-person workshop, the team developed a framework for assessing the maturity levels of people, processes, and technology. Implementation was further evaluated across six implementation factors, each with an assigned weighting of impact on the people, processes, and technology. These can be compared to the baselines established through research to indicate probable successes or failures in the technology implementation efforts at state DOTs.

This research identifies the maturity levels in the domains of people, process, and technology that a state DOT needs to achieve for probable success in technology implementation. In contrast, the research also sets the baseline levels below, which may lead to a probable failure in technology implementation. The findings enable state DOTs to self-assess their current maturity levels, identify the areas needing improvement, and use the associated six implementation factors to develop an improvement plan to reach the levels required for probable success in implementing technology.

## 2. Literature Review

Despite rapidly advancing technologies, the highway construction and asset management sectors have seen different productivity gains than other industries. Construction exhibited an alarmingly low productivity growth of only 1% over the last two decades compared to other industries. A direct correlation exists between the extent to which an industry is digitalized and its productivity growth. Unsurprisingly, construction is one of the least digitalized industries. For instance, many wireless technologies have been shown to benefit the architecture, engineering, and construction (AEC) industries. Some benefits include incremental improvements in productivity, quality and efficiency, environmental friendliness and sustainability, safety, and a reduction in waste [3]. These technologies include radio-frequency identification (RFID), the global positioning system (GPS), global navigation satellite system (GNSS), geographic information system (GIS), unmanned aerial systems or vehicles (UAS/UAV), ground-penetrating radar (GPR), lightning detection and ranging (LiDAR), e-ticketing, and others. An effective technology is one that can solve a current problem or inefficiency rather than looking for a problem to solve. The FHWA EDC initiatives promote the use of various advanced and emerging technologies (i.e., automated machine guidance, unmanned aircraft systems, building information modeling, handheld instruments and devices, and work zone intrusion detection systems).

First, RFID is an example of a technology that has long shown promise for improvements in construction and asset management but has seen little consistent implementation in the highway sector. In 2003, Jaselskis et al. proposed that RFID could be used in three primary applications in the construction industry: monitoring concrete deliveries, tracking workers and equipment, and managing critical materials. Still, many research efforts are being conducted in collaboration between academics and the industry to find the appropriate applications for RFID in highway construction [4]. Ren et al. (2011) presented an RFID-facilitated construction material management system case study for a water supply project [5]. Lodgher et al. (2010) determined and studied the feasibility of using RFID-based systems to manage assets in the state-owned right-of-way. These assets included public utilities, outdoor advertising, right-of-way markings, signs and traffic signals, bridges, culverts, buried structures (e.g., foundations, piers), fiber optic lines, tolls, and non-cut zone controls [6]. Valero et al. (2015) mentioned different uses of RFID, analyzing the various available studies that showed its uses to include: planning and design phases in the construction industry, construction components in manufacturing and the supply chain, construction, and commission control using RFID technologies, tracking systems for materials and resources, construction site monitoring, navigation, and others. These authors provided a clear explanation of the potential uses and benefits of integrating RFID systems with different technologies in construction. The article also mentions several limitations and issues that need to be addressed to make RFID accessible and usable among various stakeholders [7].

Technologies have also been shown to be useful in construction and asset management when used in combination. Hubbard et al. (2015) studied the use of both RFID and UAVs in material tracking to improve productivity by identifying the location of the materials for construction crews. The combination of RFID and UAVs allowed project managers to not only track materials but also facilitate better site management, real-time site visualization, and enhanced safety in hazardous construction sites [8]. Poor material management is one of the many reasons for slow productivity in the construction industry. The lack of active, accurate, and integrated information flow from material planning, inventory, and site use monitoring and control is a significant source contributing to the low productivity and poor management of construction projects [5]. While RFID and UAVs represent technologies to be considered, many others have had similar long-term paths to widespread use.

Harper et al. (2020) explored instrumentation and sensor technologies for highway design and construction projects. The work was based on an extensive literature review, surveys, and case studies of state DOTs. The results showed that 31 state DOTs use instrumentation and sensor technologies to monitor work progress, conduct quality control and quality assurance, perform construction inspections, identify optimal conditions, record work placement, and locate utilities. Some of the technologies discussed in the paper are remote sensing, real-time kinematic (RTK) GPS devices, ground-penetrating radar, intelligent compaction, and thermal profiling [9].

The Highway Practice Study on Emerging Technologies for Construction Delivery in 2019, sponsored by the American Association of State Highway and Transportation Officials (AASHTO) and FHWA, explored the use of emerging technologies in highway construction through a coordinated program to achieve a maximum DOT response and participation. Using technologies allows for managing resources more efficiently to complete design and construction tasks. However, using innovative technologies relies on new knowledge and skills for efficiency and the realization of the full potential of the technologies. The synthesis includes reports on the current knowledge and practice of different DOTs and the available literature. One of the three research gaps and further studies suggested in the comprehensive synthesis study includes investigating the skills and knowledge needed to use/implement technologies successfully and developing a formal framework for state DOTs [10]. The research presented herein addresses this gap by providing a detailed yet simple framework for DOTs to implement technologies successfully.

Previous research has investigated various technologies and their applications in highway construction and asset management [10,11]. However, the implementation and identification of critical implementation factors by state DOTs have been explored on a very limited basis. This presents an issue where DOTs are increasingly attracted to new emerging technologies but lack clear knowledge/guidelines on technology implementation, resulting in a higher number of unsuccessful technology implementations.

As presented, many studies have supported the use of technology in highway construction and asset management. Studies have shown that the level of success for these efforts varies [12], indicating a need to understand the factors for successful technology implementation better. Any attempt to implement technology that focuses solely on technology is likely to fail in the construction industry; therefore, combining people, processes, and technology is the central idea for building a holistic framework [13]. A successful implementation plan begins with defining success for the project, determining the greatest risks to that success, and identifying the most common factors required for successful implementation. Interestingly, less than 10% of the implementation failures resulted from technical problems; unsuccessful implementation tends to be due to human and organizational reasons [14]. Other research states that 80% of the successful implementation of new technology depends on addressing personnel and process issues, and only 20% is related to addressing technical aspects [15,16,17]. Technological innovation is important, but it should be blended with other types of innovation, such as business process and organizational innovation, to achieve ultimate success [3]. Therefore, from these and other studies, it is theorized that people, processes, and technology maturity are predictors of technology implementation success [12,13,14,15,16,17].

The people, process, and technology (PPT) framework is an established model developed in the early 1960s. Business management scholar and expert Harold Leavitt coined the original model, which comprises four elements: people, structure, tasks, and technology [18]. The PPT framework maps the entire value stream of people, processes, and technology and highlights the interactions between them: people do the work, processes make this work more efficient, and technology enables people to perform efficiently and automates the process. This framework achieves harmonization within an organization and is mainly used when implementing new technologies [18]. Therefore, for successful technology implementation, the three dimensions of people, process, and technology must be at a maturity level to support implementation success. This led to the development of maturity models to evaluate these three dimensions. Maturity models enable organizations to audit and benchmark the assessment results, reach their desired level, and evaluate elements of the organization by sequencing the maturity levels from basic to advanced stages [19].

State DOTs can benefit from these already established maturity models that assess the three dimensions noted for successful technology implementation. The benefit of using existing maturity models is in the efficient application of the lessons learned and experience. Adopting existing models and modifying them to meet certain needs is an established practice in highway construction. For example, the *National Cooperative Highway Research Program (NCHRP) Web-Only Document 214* presents a modified Capability Maturity Model to meet the study objectives of testing the feasibility of a self-assessment data program and providing guidance to implement self-assessment data methods [20]. Likewise, NCHRP Project 08-36, Task 100, modified the Capability Maturity Model to propose a framework to help transportation agencies assess their data programs [21]. The study presented here uses a similar approach to modify three established maturity models for people, processes, and technology. These established and reputable models are adapted for construction and asset management within transportation agencies. These models are used in highway construction and other fields, such as supply chain management, financial portfolio management, knowledge retention, and more. Standard maturity models have also been used in developing the BIM Maturity Index. These maturity models each have five distinct levels, and the progression from a lower to higher maturity level equates to greater effectiveness [22].

The models used for this study are detailed below as follows:

People Capability Maturity Model (PCMM): The People Capability Maturity Model is a maturity framework that focuses on continual improvement in managing and developing an organization’s human assets. It outlines an evolutionary path of improvement from inconsistently implemented ad hoc practices to complete, disciplined development and continuous improvement in the workforce’s knowledge, skills, and motivation to improve strategic business performance [23]. The PCMM guides organizations in improving their organizational processes for workforce management and development. The PCMM comprises five development levels that establish the progressive foundations for consistently improving individual abilities, creating compelling groups, propelling improved execution, and forming the labor force according to the association’s needs to achieve its future field-tested strategies [23]. The five levels of the PCMM are:

- Level 1: Initial; inconsistent management.
- Level 2: Managed; people management.
- Level 3: Defined; competency management.
- Level 4: Predictable; capability management.
- Level 5: Optimizing; change management [24].

Project Management Process Maturity Model (PM)**^2^**: The (PM)^2^ was developed by integrating previous models that measure the project management (PM) maturity levels of different companies and industries. The model assesses the position of an organization’s current PM maturity level. It illustrates a series of steps to help an organization incrementally improve its overall PM effectiveness [25]. The (PM)^2^ model integrates previous PM practices, processes, and maturity models to improve PM effectiveness in the organization incrementally. The following five levels are presented in the (PM)^2^ found in the article *Project Management Process Maturity Model*, written by Kwak and Ibbs:

- Level 1: Initial—basic PM process.
- Level 2: Planned—individual project planning.
- Level 3: Managed at the project level—systematic project planning and control.
- Level 4: Managed at the corporate level—integrated multi-project planning and control.
- Level 5: Continuous learning—continuous PM process improvement [25].

Capability Maturity Model Integration (CMMI): The Capability Maturity Model Integration (CMMI) is a process and behavioral model that helps organizations streamline their process improvement and encourage productivity [24]. The Software Engineering Institute at Carnegie Mellon University developed the CMMI with the Department of Defense and the U.S. Government [26]. The CMMI framework for defining technology maturity includes the following five maturity levels:

- Level 1: Initial—Processes are unpredictable, poorly controlled, and reactive.
- Level 2: Managed—Processes are characterized by projects and are often reactive.
- Level 3: Defined—Projects tailor their processes to the organization’s standards.
- Level 4: Quantitatively managed—Processes are measured and controlled.
- Level 5: Optimizing—Focus on process improvement [26].

These three models were chosen for this study based on (1) their use in previous studies (NCHRP Project 08-92, NCHRP Project 8-36/Task 100) and (2) their measurement alignment along a systematic five-level scale, which brings uniformity to assessing the maturity across each spectrum of people, processes, and technology.

In addition to the maturity models, a panel of experts who developed the problem statement for a recent technology implementation project (NCHRP 03-140) identified five factors as the metrics for implementing technologies at DOTs. This panel, which included individuals from the Federal Highway Administration (FHWA), DOTs, academia, and other industries, identified Organization Structure, Information Technology (IT) Infrastructure, Data Security, Information Workflow, and Personnel Training as important factors of technology implementation [27]. Later, the panel and associated researchers identified Stakeholder Engagement as the sixth important factor for technology implementation at state DOTs. 

These six factors attempt to address challenges that state DOTs face in technology implementation, some of which were mentioned by state DOT officials in the *NCHRP Synthesis Report 582*. These challenges include a lack of training, knowledge, and skills to use technologies, requirements for device maintenance and user support, a lack of reliable internet connection in remote locations, resistance to change among end-users of the technology, concerns about the quality of collected data, access, privacy, or security, incompatibility with existing hardware, insufficient agency network levels, IT infrastructure, and others [28]. The coupling of these six factors and the presented maturity models provides a foundation for developing this study.

## 3. Research Methodology

The research methodology for this study relied on the use of well-established maturity models and previously identified factors for implementation as the foundation for the developed assessment. These models and factors were adapted under the guidance of a panel of experts experienced in technology implementation in construction and asset management at DOTs. The adaptations described below were further influenced and validated by a survey of state DOT professionals and an in-person workshop with a second group of industry experts. The survey and workshop questionnaires were used to assign rankings to the identified implementation factors and their relative importance to people, processes, and technology maturity. Further, the data gathered was used to establish the baseline maturity levels and limits in experiences of both successful and unsuccessful technology implementation. This methodology is further detailed below.

After reviewing the existing maturity models for people, process, and technology, descriptions of each maturity level for the PCMM, (PM)2, and CMMI were adapted to fit the objective of this study by modifying the model terminology to the requirements of technology implementation for state DOTs regarding construction and asset management. The three maturity models are referred to hereafter as maturity dimensions. Table 1 lists the five levels used for each dimension, and the modified description of each level is provided afterward.

The five levels for People maturity using the People Capability Maturity Model (PCMM) were defined and modified for this research as follows:

- Level 1: Initial—DOTs do not have enough talented human resources required to handle projects and cannot retain qualified employees.
- Level 2: Managed—DOTs provide a good working environment and training to empower staff and provide a clear line of communication within units.
- Level 3: Defined—DOTs onboard the proper people in the proper position based on competency, experiences, and roles and responsibilities, which are well-defined and developed across an organization-wide infrastructure.
- Level 4: Predictable—DOTs have confidence in their employees and delegate tasks to empowered groups. Managers operate at higher levels with the ability to focus on more strategic issues.
- Level 5: Optimizing—The entire DOT is focused on continual improvement, including the improvement of individuals and the improvement of units for the betterment of the overall organization while focusing on central organizational objectives.

Similarly, the five levels of Process were defined and modified for this research project using the Project Management Process Maturity Model (PM)^2^ as follows:

- Level 1: Initial—DOTs understand and establish a basic project management process.
- Level 2: Planned—DOTs plan projects on individual processes and are not team oriented.
- Level 3: Managed at the project level—DOTs provide informal project management training and manage projects based on the available systems with few team members.
- Level 4: Managed at the corporate level—DOTs provide formal project management training, and multiple projects are integrated and planned with maximum team participation.
- Level 5: Continuous learning—DOTs fully understand and implement the project management procedures to create dynamic and energetic organizations that are able to manage complex projects into the future.

Finally, the five levels of maturity for Technology were defined and modified to fit the DOT setting for this research project using the Capability Maturity Model Integration (CMMI) model as follows:

- Level 1: Initial—DOTs have access to technologies but are not managed properly.
- Level 2: Managed—DOTs’ staff use technologies on a limited number of projects.
- Level 3: Defined—DOTs use and implement technologies in many projects.
- Level 4: Quantitatively managed—DOTs fully use technologies in all possible projects throughout the state organization, in the appropriate applications, and manage them properly.
- Level 5: Optimizing—DOTs fully implement technologies and identify opportunities to implement them in other projects and areas, collaborate on technologies, and update their implementation plan as needed.

This research proposes that successful technology implementation may be more likely once the appropriate maturity levels are achieved for the people, process, and technology dimensions. The definitions within the maturity models above were adapted to the metrics for DOT technology implementation. In order to support these adaptations, a survey was developed under the guidance of research team members with DOT experience with the survey and then reviewed for institutional compliance. The survey was entered into Qualtrics to allow for its digital completion and was piloted by team members with DOT experience. A definition of each maturity level was provided in the survey to ensure that all participants had the same understanding of the five levels within each dimension. An explanation of successful and unsuccessful technology implementation was briefly described but intentionally left subjective to the opinion of the subject-matter experts who would be completing the survey because it was believed specific success metrics would limit the application of the results.

The survey was sent to the American Association of State Highway and Transportation Officials (AASHTO) Committee on Construction, the AASHTO Committee on Maintenance, the AASHTO Committee on Data Management and Analytics, the AASHTO Committee on Innovation Initiative, the AASHTO Committee on Knowledge Management, and the AASHTO Subcommittee on Asset Management. AASHTO is a nonprofit, nonpartisan association representing highway and transportation departments in all 50 states, the District of Columbia, and Puerto Rico. These committee members are middle to late-career state DOT experts and are often involved in piloted approaches and technology implementation. They would further have a similar understanding of technology implementation success as resulting in a lasting change to practice.

The respondents were asked to assess the maturity levels of each dimension (i.e., people, process, and technology) for two scenarios: successful technology implementation and unsuccessful technology implementation. The respondents were also asked to elaborate on their state’s DOT experience regarding one successful and one unsuccessful technology implementation effort. This allowed the researchers to gauge the implied understanding of success in technology implementation. The respondents were then asked to rank the identified six factors of technology implementation based on their importance and provide weights of their impact on the people, process, and technology dimensions. The sum of these people, process, and technology weights was controlled by having them sum to 100%.

The maturity assessment results, rankings of the six factors, and the weights of the people, process, and technology dimensions were aggregated for each model, establishing the maturity boundaries for successful and unsuccessful technology implementation at state DOTs. While the maturity levels are considered discrete, they were averaged during the aggregating process and then rounded to the nearest maturity level boundary. After the initial analysis, the survey results were validated in an in-person workshop of state DOT experts. The workshop included 18 state DOT middle to late-career professionals. These workshop attendees were selected for their experience with implementing technologies at their respective DOT. The workshop feedback was collected through a questionnaire mirroring the digital survey previously discussed. The compiled results were then incorporated into a tool that state DOTs can use to self-assess their maturity levels across the people, process, and technology dimensions when formulating a technology implementation plan.

## 4. Results

A power analysis was conducted to determine the necessary sample size, which revealed that a minimum of 12 survey participants were needed. The results included feedback from 89 participants from the pool of AASHTO committee members, which exceeded the minimum requirements and allowed researchers to conduct more robust statistical analyses with increased precision. Collected data were checked for reliability and validity before the analysis. The internal consistency of the questionnaire items was evaluated using Cronbach’s alpha coefficient. The results indicated a moderately high level of reliability with a Cronbach’s alpha coefficient of 0.7485 (*n*= 89). The sample is further considered appropriate, as each state DOT is represented. The professionals focused on construction and asset management implementation, and the responses indicated repetition and saturation. The results were further corroborated by the second group of experts during the validation workshop. Each survey respondent was asked to identify: (1) the people, process, and technology maturity levels that lead to successful technology implementation; (2) the people, process, and technology maturity levels that result in unsuccessful technology implementation; (3) the ranking of the six implementation factors; and (4) the weight of people, process, and technology for each of these six factors. The average maturity level was then computed for each dimension (people, process, and technology) for each scenario (successful and unsuccessful technology implementation).

**Figure 1:**
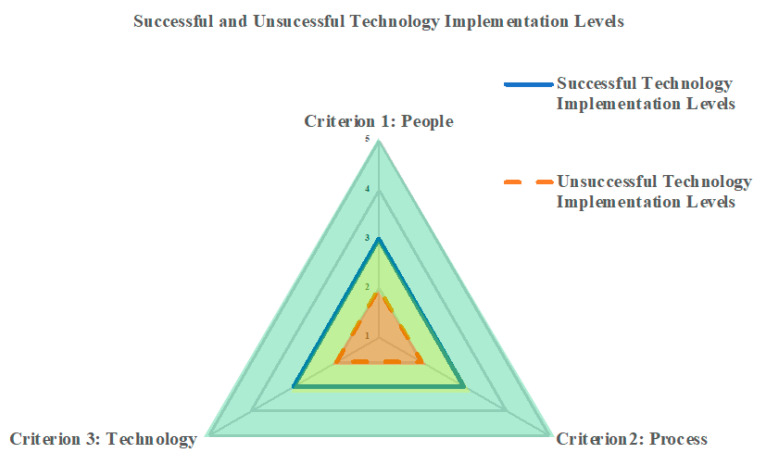
Levels of People, Process, and Technology for both successful and unsuccessful technology implementation.

Figure 1 illustrates the results for both scenarios by displaying: (1) the maturity level required for each dimension to achieve a successful technology implementation (solid line) and (2) the maturity level for each dimension below, which results in an unsuccessful technology implementation (dashed line). From the results and validation, state DOTs seeking successful technology implementation should achieve at least a level 3 maturity for people (“defined” (average value of 3.25)); at least a level 3 maturity for the process dimension (“defined” (average value of 3.34)); and at least a level 3 maturity level for technology (“defined” (average value of 3.31)). The findings also show that unsuccessful technology implementation can be expected when a state DOT is at or below a level 2 (“managed” (average value of 1.92)) maturity level for people at or below a level 2 (“managed” (average value of 2.20)) maturity level for process, and at or below a level 2 (“managed” (average value of 2.21)) maturity level for technology. The two lines in Figure 1 define the boundaries for successful and unsuccessful technology implementation. The red area represents the maturity levels of people, processes, and technology at or below the dashed line, likely yielding an unsuccessful technology implementation effort. The green area represents maturity levels at or above the solid line, representing probable success in implementing technology. Furthermore, the yellow area represents the maturity levels between successful and unsuccessful implementation, indicating an area that still likely needs improvement.

The state DOTs can use this assessment to understand where their efforts need to be expended to improve the likelihood of technology implementation success, either in the maturity of people, process, technology, or some combination thereof.

In a second segment of the survey, the respondents (*n* = 74, which is still considered an appropriate sample based on the previously described conditions) provided a rank order for the six implementation factors of Organization Structure, IT Infrastructure, Data Security, Information Workflows, Personnel Training, and Stakeholder Engagement. The ranking of “1” was used to indicate factors of the highest importance. The responses were categorized, and a weighted average was calculated to find the overall ranking of the implementation factors. Table 2 provides a summary of the response rankings.

**Figure 2:**
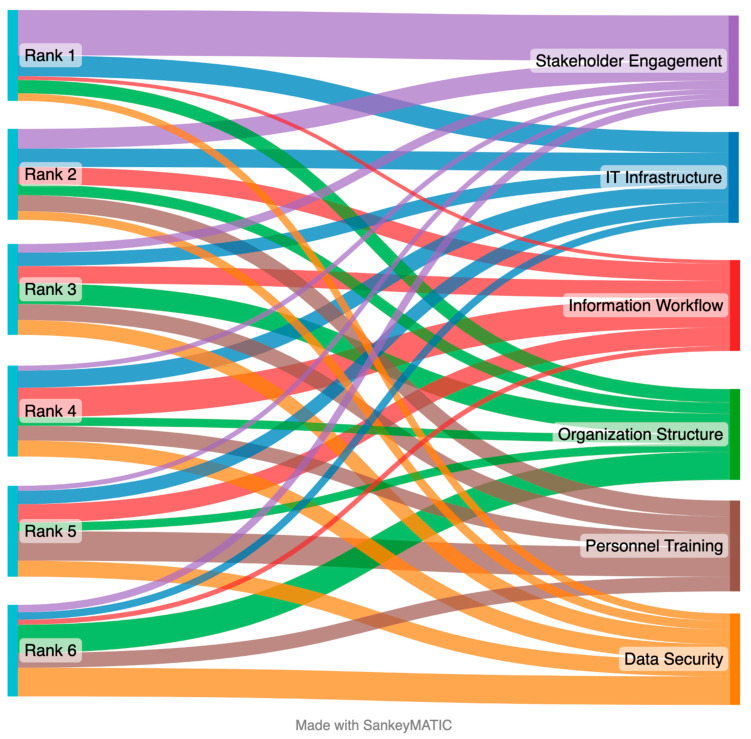
Ranking of six technology implementation factors using the Sankey diagram (*n* = 74).

These responses are represented as the bandwidth in Figure 2. This figure shows the number of respondents who selected a particular rank for each technology implementation factor. The width of the flows from the rankings to the technology implementation factors is based on the number of responses for that respective segment. The thicker width of the flow means a higher number of responses made in that selection, and a thinner width of the flow means fewer responses for that connected rank-factor pair. The overall ranking order of the six technology implementation factors, as calculated by weighted average, is Stakeholder Engagement, IT Infrastructure, Information Workflow, Organization Structure, Personnel Training, and Data Security. While Figure 2 illustrates some response variation, the weighted average presents the compiled order of importance for the implementation factors.

**Figure 3:**
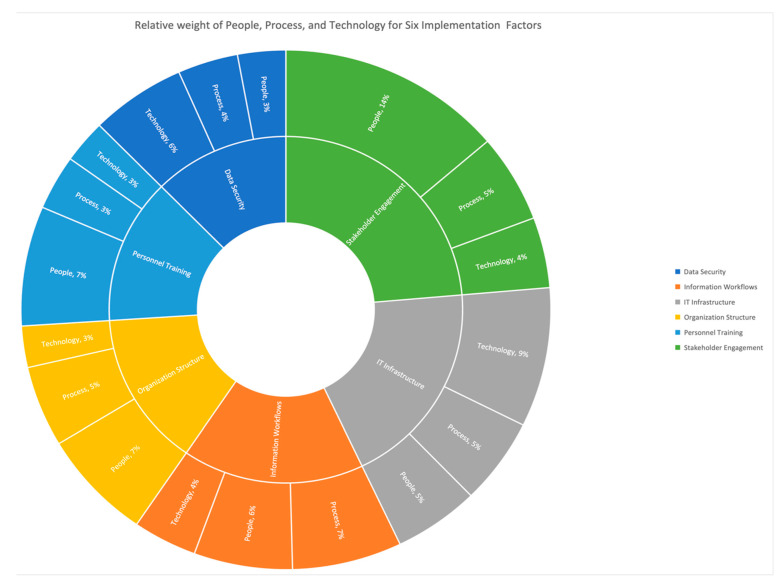
Relative weights of People, Process, and Technology for six factors of technology implementations.

In the final segment of the survey, the respondents were asked to provide the weight of importance of people, process, and technology for each of the six implementation factors. As mentioned, these weightings were controlled to a total of 100%. By using these responses, the average weights of the people, process, and technology dimensions were calculated for each implementation factor. The results from the survey are illustrated here in Figure 3 for each implementation factor but as a relative weight. The relative weight was calculated as the product of the weight of an implementation factor (determined from the rankings) and the individual weight responses for people, process, or technology of that same implementation factor.

Following the survey, the results were validated using an in-person workshop of 18 transportation subject-matter experts. Table 3 and Table 4 present the comparisons of the average maturity levels from the survey and validation workshop for the successful and unsuccessful implementation of technology. Comparing the maturity level results reveals nearly identical average maturity levels for successful and unsuccessful technology implementation. The results align with the same limit as identified in Figure 1.

**Figure 4:**
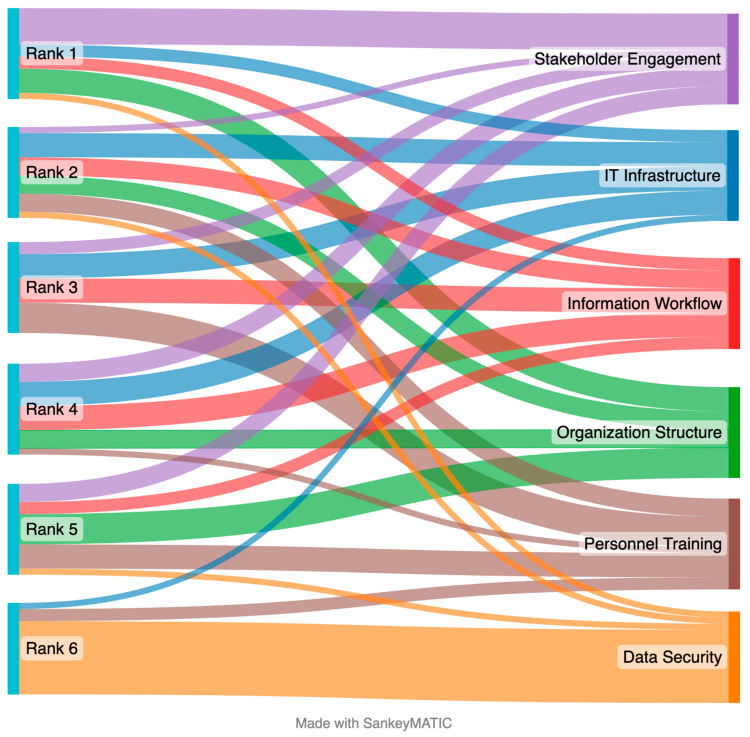
Ranking of six technology implementation factors for workshop validation data using the Sankey diagram (*n* = 15).

The implementation rankings from the survey data were validated through feedback received during the in-person workshop. Fifteen complete validation responses were collected and categorized, and a weighted average was calculated to determine the overall ranking of the implementation factors. Table 5 summarizes the respondent rankings, which were also used to create the diagram in Figure 4. The compiled ranking order of the six technology implementation factors was the same for the survey and validation workshop results.

The workshop participants were also asked to provide weights of importance for the dimensions of people, process, and technology for each implementation factor. Comparing these weights from the survey and the workshop revealed almost identical results for each technology implementation factor. Table 6 compares the average weight of the people, process, and technology dimensions for the six factors of technology implementation.

**Figure 5:**
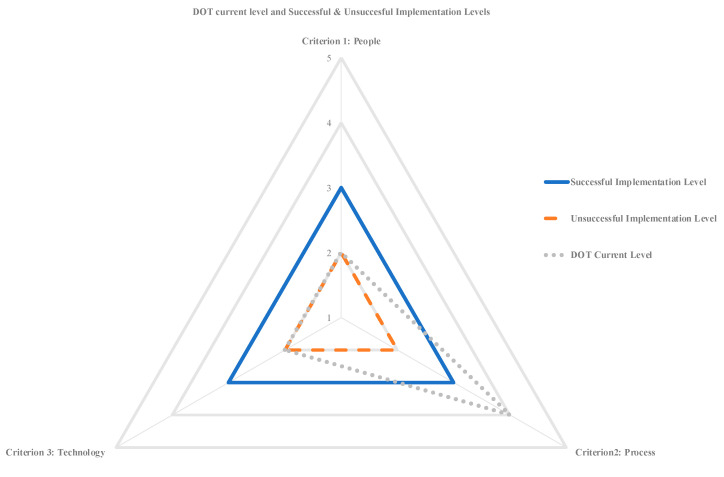
DOT current level, successful, and unsuccessful technology implementation levels visualization.

With the results validated, the information was seemingly appropriate for use by state DOTs to conduct self-assessments to determine their current maturity levels along the dimensions of people, process, and technology and compare these with the maturity limits established through this research. The approach could follow the concept illustrated in Table 7. Table 7 displays the model’s five levels for each dimension (people, process, and technology) and allows the state DOT to select its current maturity level (seventh column). The maturity boundaries established through this research for each dimension are available in columns 8 and 9. Once the user has selected their state DOT’s current maturity level, a chart similar to Figure 5 shows the maturity boundaries and the current assessment of the state DOT’s maturity levels (represented by the dotted line).

Table 7 and Figure 5 enable state DOTs to identify their current maturity levels and contemplate the probability of the success of their technology implementation effort. The table and figure also allow the state DOT to discern where improvements are needed for an improved likelihood of technology implementation success. The levels selected in Table 7 are provided for illustrative purposes but point to the need for the example state DOT to consider improvements along People and Technology to have an improved chance of successful technology implementation.

## 5. Discussion

This research investigates the people, process, and technology dimensions as major contributors to the success of technology implementation efforts. A state DOT collective of maturity was found to be a distinguishing factor in the success or failure of technology implementations. To better understand the interplay between these three dimensions, the survey respondents were asked to explain how people, processes, and technology collectively contributed to a successful technology implementation effort or resulted in an unsuccessful technology implementation effort.

The analysis of this collected data showed that successful technology implementations involved good coordination between the people, process, and technology dimensions. One respondent provided the following explanation of how their state DOT leveraged these three dimensions to achieve success in implementing technology:

“The very first step is to understand the current business processes/standards established by agency guidelines/standards/processes. Once these are identified, the project/technology is tailored to improve or suit the identified guidelines/standards/processes. A core team of employees is selected as part of the implementation team based on experience and capabilities. The core team manages the project at a project level, with occasional inputs from experts or management when needed. This process allows for flexibility at the project level and technology adaptation to the current business processes/standards to avoid any unintended interruptions.”

Other respondents noted that their state DOT adopted a proactive approach and developed research capabilities where technologies are thoroughly vetted before implementation. These responses align with the approach presented herein.

Analysis of the responses to the unsuccessful technology implementation efforts showed an increased emphasis on technology while neglecting the people dimension as a common theme among those experiencing technology implementation failures. Moreover, the respondents also noted that, while the people and process dimensions might be mature, when technology maturity is low, such as when the technology is not compatible with existing systems, a failure is imminent. This emerged as a critical challenge, especially when the technology was driven from outside the organization, and the state DOT did not have the right processes in place to train their employees or understand their needs. Additionally, the implementation efforts were often challenged when stakeholders were not engaged. These examples included a lack of end-user input, lack of personnel training at roll-out, no direct support and commitment from the leadership, and unaddressed concerns or no long-term support. It was found that the difference between the successful and unsuccessful implementation of technology is conditioned upon the presence of the right people having the right mindset and working together in preparation for the needs of technology and processes. The probability of success in technology implementation will improve by increasing the levels of maturity across the people, process, and technology dimensions, whereas an unsuccessful implementation will typically have maturity below the baselines previously described.

The rankings identified the six factors of technology implementation that would help state DOTs focus on areas of importance. It is worth noting that each state DOT has different capabilities, but having the references identified herein will guide them to areas of needed improvement. The weights of the people, process, and technology dimensions for each of the six factors are important findings and provide more resources for DOTs when considering technology implementation. The different weights of the people, process, and technology dimensions for each factor of technology implementation show that the factors necessitate varying levels of attention to strengthen the likelihood of technology implementation success. Knowing where to focus, whether it is the people, process, or technology, will prepare state agencies for the improved potential of success in technology implementation and for solving challenges quickly and effectively. These findings can help unit management take on issues or move them to upper-level management for support. The findings will also help upper-level management to provide the required levels of support to business units within the DOT and justify any organizational policy changes and requirements.

The results indicate that state DOTs should focus on the people, process, and technology triad. These three dimensions are mutually dependent and must be aligned and in harmony. Adopting digital technologies that support transportation construction and asset management activities depends on many factors. Some digital technologies are more disruptive than others and require a specialized level of expertise and a high level of investment, in which a more strategic approach to implementation is necessary. On the other hand, technologies such as mobile devices are less disruptive and have a lower barrier to entry because they are simpler tools requiring little experience and training. Regardless of the level of disruption and complexity, all digital technologies have certain factors to consider for their successful implementation.

To summarize the implementation factors, it was found that data security, which deals with data collaboration and accessibility, data security policies, data management, and data governance, has a high weight in technology. Personnel Training deals with training strategies, delivery modes, frequency of training, and technology support, which has a high weight for people. Information Workflows deal with data collection, processing, and validation, which is highly weighted in the process dimension. IT Infrastructure deals with hardware capabilities, software capabilities, data storage, and mobility, which have a high weight for technology. Organization structure deals with organizational vision and objectives, management support, and technical oversight, which has a high weight for people. Stakeholder Engagement, which deals with external stakeholder coordination, internal stakeholder coordination, and stakeholder readiness, has a high weight for people. These weights emphasize the potential focus areas among these implementation factors and associated dimensions.

## 6. Conclusions

In conclusion, this study highlights the crucial role of people, processes, and technology in determining the success or failure of technology implementation in state DOTs. Through a survey of subject-matter experts and workshop validation, maturity limits have been identified for assessing the potential success or failure of a technology implementation effort. Additionally, the study ranks six implementation factors and their weights for the people, process, and technology dimensions. These results indicate that stakeholder engagement is the most important factor in implementing technology. This conclusion alone illustrates to state DOTs that an attempted implementation of technology without the inclusion of feedback from stakeholders and end-users will likely face challenges. The weighted importance of these combinations can provide state DOTs with a comprehensive framework to evaluate their planned technology implementations and estimate the potential success or failure of such endeavors before investing in them, thereby minimizing expenses and risks.

The findings herein align with similar works and models discussed in the literature review. As noted by experts, success in technology implementation is based on the adoption and emphasis of people who drive the processes that allow for piloting and implementing the technology. With ongoing associated research efforts, this work is expected to significantly assist state DOTs as they consider implementing technologies in highway construction and asset management.

Future research will focus on conducting more detailed investigations into each technology implementation factor, providing state DOTs with more specific guidelines to boost implementation success and productivity. The researchers also plan to validate their findings through detailed case investigations with selected state DOTs, including those who have successfully implemented technologies and those who have struggled with implementation. These cases will provide further insight into the usefulness and impacts of the research findings during technology implementation.

Noted limitations of this study include that the determination of technology implementation success is founded on a subjective measure; however, this limitation allows for broad applicability of the results. The subject-matter experts surveyed would also be the end-users of these results and have similar understandings of technology implementation success and failure. There is additional subjectivity in the approach developed within the study that involves a self-assessment along broad models. This is also necessary to provide applicability across a wide range of technologies and modes of implementation. Another limitation is the studies use only six specific technology implementation factors. While subject-matter experts identified these six factors, future studies can explore and expand this list. Finally, the research is limited by its broad nature in not focusing on a specific technology. As a result, the experiences of the survey respondents might be limited to certain technologies or state DOTs practices, thus reflecting those experiences in their survey responses.

## Figures and Tables

**Table 1:** Summary of the assessment of the three dimensions of people, process, and technology.

Maturity Dimension			
Level	People	Process	Technology
1	Initial	Initial	Initial
2	Managed	Planned	Managed
3	Defined	Managed at Project level	Defined
4	Predictable	Managed at Corporate Level	Quantitatively Managed
5	Optimizing	Continuous Learning	Optimizing

**Table 2:** Number of responses ranking the implementation factors.

Number of Respondent Rankings						
	Rank 1	Rank 2	Rank 3	Rank 4	Rank 5	Rank 6
Organization Structure	11	9	17	7	7	23
IT Infrastructure	17	15	11	14	11	6
Data Security	6	7	12	13	13	23
Information Workflows	3	14	14	24	15	4
Personnel Training	0	13	13	12	24	12
Stakeholder Engagement	37	16	7	4	4	6

**Table 3:** Average maturity levels for successful technology implementation from the survey and workshop validation.

Dimension	Maturity Level from the Survey	Maturity Level from the Workshop
People	3.26	3.27
Process	3.34	3.35
Technology	3.37	3.32

**Table 4:** Average maturity levels for unsuccessful technology implementation from survey and workshop validation.

Dimension	Maturity Level from the Survey	Maturity Level from the Workshop
People	1.94	1.89
Process	2.25	2.15
Technology	2.23	2.20

**Table 5:** Number of responses ranking the implementation factors.

Number of Respondent Rankings						
	Rank 1	Rank 2	Rank 3	Rank 4	Rank 5	Rank 6
Organization Structure	4	3	0	3	5	0
IT Infrastructure	2	4	4	4	0	1
Data Security	1	1	0	0	1	12
Information Workflows	2	3	4	4	2	0
Personnel Training	0	3	5	1	4	2
Stakeholder Engagement	6	1	2	3	3	0

**Table 6:** Weight of People, Process, and Technology from the survey and workshop validation.

Implementation Factor	Criteria	Weight from Survey	Weight from Validation
Data Security	People	24%	25%
	Process	30%	30%
	Technology	47%	45%
Information Workflows	People	36%	34%
	Process	40%	43%
	Technology	24%	24%
IT Infrastructure	People	27%	28%
	Process	28%	28%
	Technology	45%	44%
Organization Structure	People	47%	46%
	Process	35%	35%
	Technology	18%	18%
Personnel Training	People	55%	55%
	Process	25%	25%
	Technology	20%	19%
Stakeholder Engagement	People	58%	57%
	Process	23%	24%
	Technology	19%	19%

**Table 7:** Self-assessment of state DOTs for Technology implementation.

Criteria	Level 1: Initial	Level 2: Managed	Level 3: Defined	Level 4: Predictable	Level 5: Optimizing	Your DOT Level	Successful Impl. Level	Unsuccessful Impl. Level
People	DOTs do not have enough talented human resources required to handle projects and cannot retain qualified employees.	DOTs provide a good working environment, training to empower staff, and a clear line of communication within units.	DOTs inboard the right people in the right position based on competency, experiences, and roles, and responsibilities are well-defined and develop an organization-wide infrastructure.	DOTs have confidence in employees and delegate tasks to empowered groups. The managers at higher levels are able to focus more on strategic issues.	The entire DOTs are focused on continual improvement and improvement of individuals to the improvement of units to the improvement of the overall organization while focusing on main objectives.	Level 2	3	2
Process	DOTs understand and establish basic project management processes.	DOTs plan the projects on individual processes and are not team oriented.	DOTs provide informal project management training and manage project based on available systems with few team members.	DOTs provide formal project management training, and multiple projects are integrated and planned with maximum team participation.	DOTs fully understand and implement project management procedures to create dynamic, energetic organizations and manage complex projects in the future.	Level 4	3	2
Technology	DOTs have access to technologies, and technologies are not managed properly.	DOTs staff using technologies only in a handful of projects	DOTs use and implement technologies in many projects.	DOTs fully use technologies in all possible projects throughout the state for appropriate applications and are managed properly.	DOTs fully implement technologies and find ways to implement them in other projects, technologies collaboration, and updating their implementation plan as required	Level 2	3	2

## Data Availability

The data presented in this study are available on request from the corresponding author.
